# Examining the reliability of brain age algorithms under varying degrees of participant motion

**DOI:** 10.1186/s40708-024-00223-0

**Published:** 2024-04-04

**Authors:** Jamie L. Hanson, Dorthea J. Adkins, Eva Bacas, Peiran Zhou

**Affiliations:** 1 Learning, Research & Development Center, University of Pittsburgh, Murdoch Building 3420 Forbes Ave. Rm. 639, Pittsburgh, PA 15260 USA; 2 Department of Psychology, University of Pittsburgh, Pittsburgh, PA USA

**Keywords:** Brain age, Neuroimaging, MRI, Motion artifacts, Image quality, Algorithm Reliability, Biomarker

## Abstract

Brain age algorithms using data science and machine learning techniques show promise as biomarkers for neurodegenerative disorders and aging. However, head motion during MRI scanning may compromise image quality and influence brain age estimates. We examined the effects of motion on brain age predictions in adult participants with low, high, and no motion MRI scans (*Original N* = *148; Analytic N* = *138*). Five popular algorithms were tested: brainageR, DeepBrainNet, XGBoost, ENIGMA, and pyment. Evaluation metrics, intraclass correlations (ICCs), and Bland–Altman analyses assessed reliability across motion conditions. Linear mixed models quantified motion effects. Results demonstrated motion significantly impacted brain age estimates for some algorithms, with ICCs dropping as low as 0.609 and errors increasing up to 11.5 years for high motion scans. DeepBrainNet and pyment showed greatest robustness and reliability (ICCs = 0.956–0.965). XGBoost and brainageR had the largest errors (up to 13.5 RMSE) and bias with motion. Findings indicate motion artifacts influence brain age estimates in significant ways. Furthermore, our results suggest certain algorithms like DeepBrainNet and pyment may be preferable for deployment in populations where motion during MRI acquisition is likely. Further optimization and validation of brain age algorithms is critical to use brain age as a biomarker relevant for clinical outcomes.

**Supplementary Information:**

The online version contains supplementary material available at 10.1186/s40708-024-00223-0.

## Introduction

Recent advances in machine learning and data science have led to a number of research projects focused on the concept of “brain age”. This novel metric uses large datasets where age and neuroimaging scans are available, and age can be estimated on new participants only using neuroimaging data (for review, see [1]). Interestingly, these neuroimaging estimates can diverge from participants' chronological age, suggesting potential alterations in an individual’s biological age. An increasing focus on brain age is in part due to its promise as a potential biomarker for neurodegeneration, cognitive decline, and multiple psychiatric issues (e.g., schizophrenia, major depression, bipolar disorder; [2]). Given that many of these conditions are highly prevalent, early detection, via brain age or other metrics, could have important public health implications.

While potentially powerful, numerous open questions exist regarding brain age, especially in thinking about use of this metric in clinical settings. Of particular importance is how noise and image quality may influence the derivation of brain age. Noise and poor image quality arises, in part, from participant head movement during an MRI scan. Notably, high levels of head motion have been found in children, older adults, and clinical patient groups, as compared to young adult, non-patient samples [3–6]. High rates of head motion can eventually lead to erroneous estimates of cortical thickness, surface area, and volume [7–9]. This has been noted in both adult [8, 10], as well as pediatric samples [11]. As such, head motion during imaging sessions may influence our ability to detect differences in brain age between different groups, or in relation to behavioral traits of interest [12].

Pursuant to the clinical utility of brain age, researchers have developed multiple algorithms to calculate brain age. While one can use multimodal neuroimaging [13], most algorithms use T1-weighted anatomical images, either processed in Freesurfer or in NIfTI format to estimate brain age [14–16]. With head motion likely to compromise measures of brain morphometry [7, 8, 17], image quality and participant motion could be introducing biases into brain age calculations. Put another way, image quality and motion could lead to lower estimates of cortical thickness, surface area, and volume, resembling the cortical atrophy associated with typical aging [18]. In this way, head motion artifacts could bias estimates of brain age, leading to accelerated brain age being inaccurately noted in high motion participants. Connected to this, work from our group has found modest correlations between raw brain age and image quality (r = − 0.38 to − 0.46), as well as between brain age gaps (the differences from raw brain age and a participant’s chronological age) and image quality (max r = 0.36, [19]).

While notable, our past work may be underestimating the true impact of motion and noise in brain age calculation. We previously examined bivariate correlations between image quality and brain age by assessing brain age and scan quality across different individuals. As such, potential relations may be occluded because of between-person variations in brain age. To understand the impact of motion and noise more deeply in brain age calculation, it will be important to examine intra-individual relations between image quality and brain age. Put another way– If we only look at between person effects, people who are able to stay still more frequently and produce higher quality MRI scans may have slower brain aging, but these individuals may also have better cognitive functioning and other factors that also relate to brain aging. As such, image quality and other third factors related to brain age collide. Such factors could be both causes and effects, and we cannot statistically separate these within our typical (between-level) study designs. To truly understand the impact of motion and noise on brain age, it would be advantageous to examine brain age within the same participants repeatedly scanned when they are remaining still and when they exhibit higher levels of head motion.

**Fig. 1:**
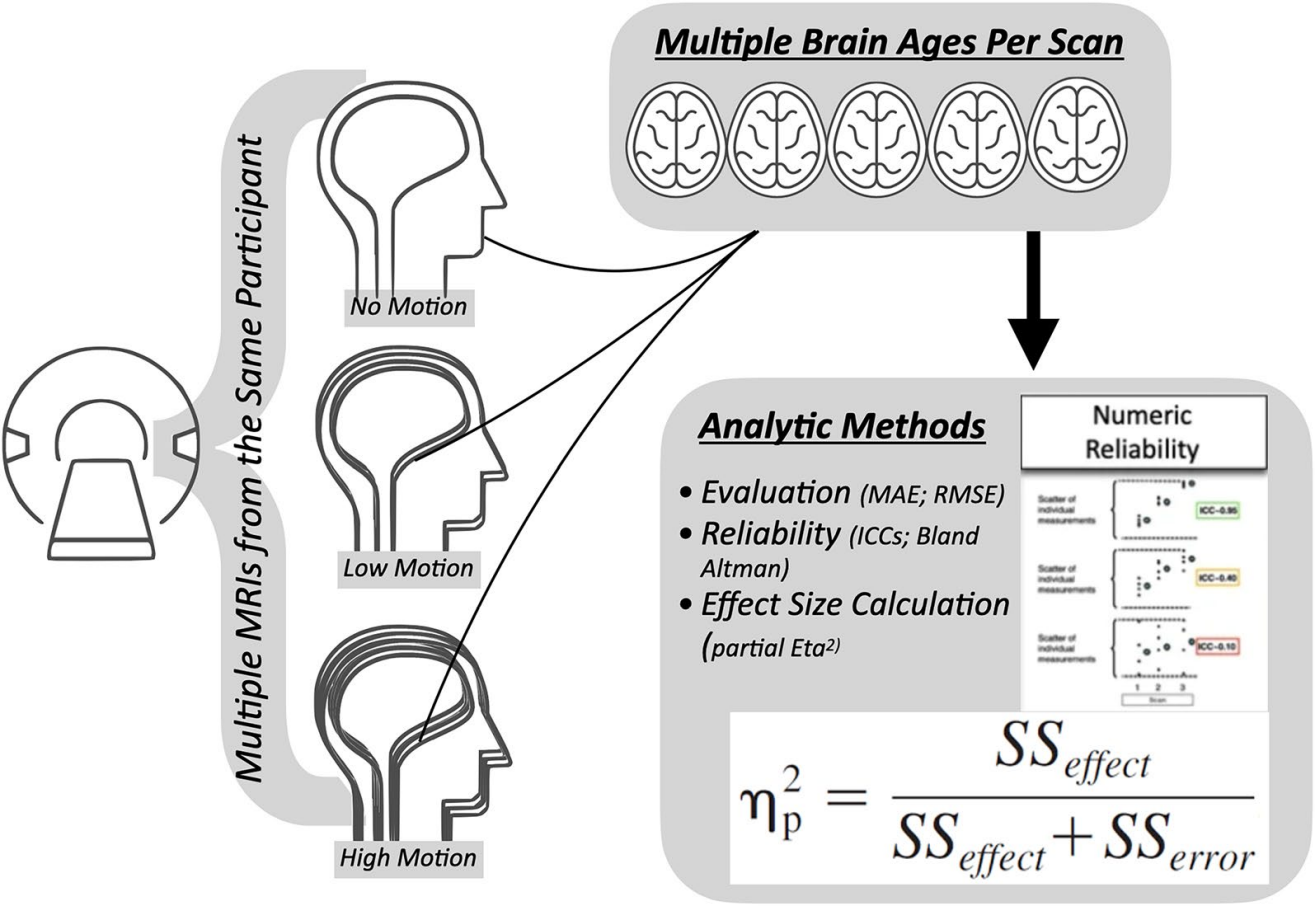
Graphical depiction of study design

Motivated by this, we leveraged a public access dataset to examine the impacts of motion on brain age. This dataset had high-, low-, and no-motion scans for the same individuals [20]. Processing these datasets through multiple commonly used brain age algorithms (2 algorithms using Freesurfer-derived outputs; 3 algorithms using less processed NIfTI images), we first calculated evaluation metrics between participants’ chronological age and brain age predicted from each algorithm (using Mean Absolute Error and Root Mean Squared Error). We then examined intraclass correlations and Bland–Altman bias metrics to compare algorithmic performance across repeated MRI scans of the same individuals (as depicted in Fig. 1). We also constructed linear mixed effect models that could accommodate repeated measures from the same individuals and examined the high-, low-, and no-motion conditions. This would allow us to derive standardized estimates for each algorithm in relation to low- and high-motion scans. Given past work finding motion and noise related to lower gray matter levels, we predicted lower correlations between chronological age and brain age for high motion scans. We also predicted algorithms that relied on Freesurfer would have greater changes in these metrics for high motion scans, since image quality is related to variations in morphometric estimates from this software [11]. Of note, we believe this is the first study to systematically examine multiple brain age algorithms on the same dataset with controlled levels of motion artifact. Our work can provide a more comprehensive assessment of brain age algorithms across multiple reliability metrics. Furthermore, by using this within-participant design with repeated scans, we believe we are able to more strongly understand the effects of head motion on brain age and more robustly control for individual differences.

## Method

### Dataset and participants

**Fig. 2:**
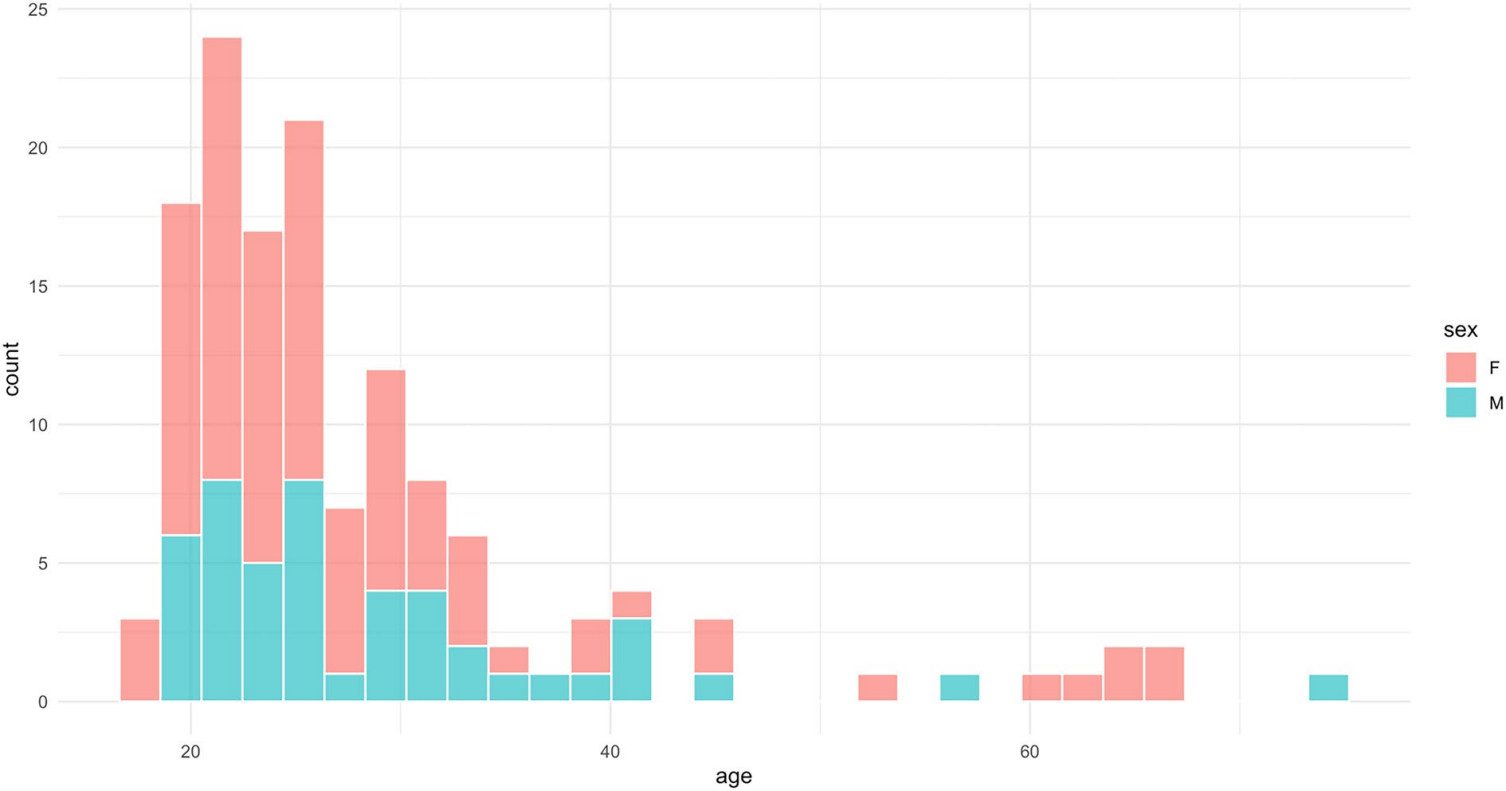
Age and sex distribution of study participants. Histogram showing the age and sex distribution of the 138 adult participants with usable data included in the study, ages 18–75 years

We examined a public-access MRI dataset that included 148 healthy adult participants, ages 18–75 years (Mean Age = 30.01 ± 12.76 years; 64.1% Female; age-by-sex histogram shown in Fig. 2) [20]. During MRI acquisition (detailed below), participants were instructed not to move at all, and in other scans to nod their head. To create different levels of motion artifacts, the word “MOVE” was presented (in Hungarian) for 5 s, 5 or 10 times evenly spaced during image acquisition. Nodding was used as a motion induction since it is reportedly the most prominent type of head motion, responsible for most MRI artifacts [21]. This procedure yielded images with minimal head motion, as well as with slight (low) and more excessive (high) head motion. Participants gave informed consent and reported no neurological or psychiatric diseases. Collection protocols were approved at the National Institute of Pharmacy and Nutrition in Hungary. These repeated scans, with varying levels of motion, allowed for rich evaluation of the impact of motion artifacts on MRI image quality and brain age calculations. Of note, the final analytic sample was 138 due to failure in preprocessing or missing scans (details noted below)

#### MRI data acquisition

The dataset includes three whole brain T1-weighted MRI images that were acquired using a Siemens Magnetom Prisma 3 T MRI scanner (Siemens Healthcare GmbH, Erlangen, Germany) with the standard Siemens 20-channel head-neck receiver coil at the Brain Imaging Centre, Research Centre for Natural Sciences. Each T1-weighted 3D magnetization-prepared rapid gradient echo (MPRAGE) anatomical image was acquired using twofold in-plane GRAPPA acceleration with isotropic 1 mm3 spatial resolution (repetition time (TR) = 2300 ms, echo time (TE) = 3 ms, inversion time (TI) = 900 ms, flip angle (FA) = 9°, FOV = 256 × 256 mm). Of note, three T1-weighted structural scans were acquired with the same parameters for each participant in a standard setting, with no  (N), low (L) and high (H) levels of head motion.

During each scan, a fixation point was presented at the center of the display, and participants were instructed to gaze at this point. For the N (no motion) scan, participants were instructed to remain completely still. In the case of the L and H scans, participants were directed to nod their heads (tilt them down and then up along the sagittal plane) once upon the appearance of the word “MOVE” on the screen. We chose nodding as the motion pattern, as it is recognized as the predominant form of head movement, associated with the majority of motion artifacts [21–26]. To introduce varying levels of motion artifacts, the word “MOVE” was displayed for 5 s, occurring 5 and 10 times evenly spaced throughout image acquisition for the L (5 times) and H (10 times) scans, respectively. Participants were explicitly advised not to lift their heads from the scanner table while nodding and were instructed to return their heads to the original position after each nod. This dataset is publicly available at OpenNeuro at: https://openneuro.org/datasets/ds004173/.

### Brain age algorithms

We deployed five brain age algorithms on this dataset: Cole et al. [14] (referred to as “brainageR”), Kaufmann et al. [15] (referred to as “XGBoost”), Bashyam et al. [16] (referred to as “DeepBrainNet”), Han et al. [27] (referred to as “ENIGMA”), and Leonardsen et al. [28] (referred to as “pyment”). We selected these algorithms based on popularity in recent brain age publications and their open access code. For pyment, DeepBrainNet and brainageR, T1-weighted MRI scans are input and preprocessed by each algorithm. In contrast, XGBoost and ENIGMA require preprocessing using Freesurfer [29], an open-source MRI processing software package. Of note, for a small number of participants Freesurfer processing was not successful due to extensive noise (N = 7 of our original sample size) or missing scans (N = 3). This left a final, usable N of 138. We provide brief summaries of the algorithms below. For detailed descriptions of model structure, please see the original papers cited here.

#### brainageR [14] brain age algorithm

brainageR loaded T1-weighted MRI images into SPM12, where these anatomical images were segmented and normalized with custom brain templates. After this, the resulting segmented and normalized images were loaded into R [14]. In R, gray matter, white matter and CSF were vectorized. The rotation matrix of a previously calculated Principal Components Analysis was then applied to gray matter, white matter and CSF vectors to predict an age value with the trained model with *kernlab* (using a Gaussian Process Regression with Radial Basis Function kernel and default hyperparameters). This algorithm (version 2.0 24 Sep 2019) was trained on a sample (N = 2001) of healthy adults aged 18–90. Relevant code is available at: https://github.com/james-cole/brainageR.

#### DeepBrainNet [16] brain age algorithm

DeepBrainNet is a 2D Convolutional Neural Network (CNN) built using the inception-resnetv2 framework and pre-trained on ImageNet [16]. With this algorithm, raw, unprocessed, T1-weighted MR images are N4 bias corrected, skull-stripped, and affine registered to an MNI-template. This algorithm was implemented through the ANTsRNet package, an implementation of Advanced Normalization Tools (ANTs) in the R programming language [30]. This algorithm was trained on a sample (N = 11,729) of healthy controls aged 3–95. Relevant code for this algorithm is located here: https://github.com/ANTsX/brainAgeR.

#### XGBoost [15] brain age algorithm

XGBoost uses gradient tree boosting to predict brain age based on 1118 features extracted using Freesurfer [15]. These features consist of thickness, area, and volume measurements from a multimodal parcellation of the cerebral cortex, cerebellum, and subcortex [31]. This algorithm was trained on a large and diverse sample (N = 35,474). Kaufmann et al. trained separate models for male and female brain age. We deployed this algorithm by first completing standard processing approaches in Freesurfer 7.1 (http://surfer.nmr.mgh.harvard.edu). This processing includes motion correction and intensity normalization of T1-weighted images, removal of non-brain tissue, automated Talairach transformation, segmentation of white matter and gray matter volumetric structures, and derivation of cortical thickness [32–36]. Freesurfer processing was implemented via Brainlife.io (brainlife/app-freesurfer), which is a free, publicly funded, cloud-computing platform for developing reproducible neuroimaging processing pipelines and sharing data [37]. The technical details of this software suite are described in prior publications. Relevant code for the XGBoost algorithm is available at: https://github.com/tobias-kaufmann/brainage.

#### ENIGMA [27] brain age algorithm

The ENIGMA algorithm used ridge regression based on Freesurfer features (processing details for Freesurfer, noted above). The ENIGMA algorithm was developed based on data from N = 2,188 participants [27]. Structural MRI measures output from Freesurfer, from both the left and right hemispheres, were combined. Specifically, this resulted in 77 brain features including subcortical volumes, cortical thickness and surface area. Normative models were then estimated in a training sample of male and female controls. Relevant code for this algorithm is located here: https://photon-ai.com/enigma_brainage.

#### Pyment [28] brain age algorithm

The pyment algorithm implemented a Simple Fully Convolutional Network on T1-weighted structural magnetic resonance images. These images were partially preprocessed in FreeSurfer (using this software’s -autorecon1 steps). Training dataset was one of the largest and most diverse datasets assembled (N = 34,285), stratified by age and study. Additional technical details are available in the original report (Ref. [28]). Relevant code for this algorithm is located here: https://github.com/estenhl/pyment-public.

### MRI image quality assessment

While head motion varied based on instructions to participants, we also quantitatively measured image quality, an indirect assessment for head motion, using the CAT12 toolbox. Specifically, we generated a quantitative metric (“CAT12 score”) using the Computational Anatomy Toolbox 12 (CAT12). This metric considers four summary measures of image quality: noise-to-contrast ratio, coefficient of joint variation, inhomogeneity-to-contrast ratio, and root-mean-squared voxel resolution. CAT12 normalizes and combines these measures using a kappa statistic-based framework. The score is a value from 0 to 1, with 0 being the lowest quality and 1 being the highest quality. This measure was used for two purposes: (1) to confirm different levels of motion artifacts of repeated scans; and (2) to allow for a more continuous investigation of the impact of image quality (and connected head motion) on brain age estimates.

Of note, quantifying and estimating participant movement is a complex area of current investigation for many. We did not have direct assessments of participant movement, which is an important issue to highlight. We, however, believe that CAT12 may be a reasonable proxy for participant movement, especially in more applied settings. In our past work, we have found that CAT12 differentiates passing human visual checks for MRI scan inclusion (receiver operating characteristic curve = 98.9%). In this past work, nearly all of the examined MRI scans were excluded for participant movement. Furthermore, CAT12 scores strongly correlate with other previously used metrics of MRI quality, Freesurfer’s Euler Number r = − 0.904. CAT12, however, outperformed Freesurfer’s Euler Number in determining scan inclusion and exclusion (as determined by human visual checks, as previously reported in [11]).

### Statistical analyses

Each brain age algorithm output a predicted brain age, which was used as a continuous variable in multiple analyses. We also calculated a brain age delta, or (predicted brain age—chronological age). This is the most commonly used metric in applied brain age studies, with higher values denoting accelerated aging. After we organized these values, our analysis included calculation of: (1) evaluation metrics (MAE; RSME); (2) Intraclass correlations; (3) Bland–Altman bias measures; (4) effect size estimates from linear mixed effect models. We sought to do this for each of these five algorithms noted above.

#### Evaluation metrics for each algorithm

We calculated differences between participants’ chronological age and brain age predicted from each algorithm. Specifically, we used Mean Absolute Error (MAE) and Root Mean Squared Error (RMSE). MAE is calculated by taking the absolute differences between predicted (brain age) and true (chronological age) values and averaging, while RMSE measures the square root of the average squared differences between predictions and true values. MAE is in the same units as the data, while RMSE is not and penalizes larger errors more heavily.

#### Brain age reliability assessment by intraclass correlations

To assess the reliability of brain age calculation by algorithm, we used two approaches of looking at reliability: intraclass correlation coefficient (ICC) and Bland–Altman analysis. ICC is a descriptive statistic indicating the degree of agreement between two or more sets of measurements. The statistic is similar to a bivariate correlation coefficient insofar as it has a range from 0 to 1 and higher values represent a stronger relation. An ICC differs from a bivariate correlation in that it utilizes groups of measurements and gives an indication of the numerical cohesion across the given group [38]. We calculated ICCs using the statistical programming language R, with the icc function from the package *“irr” *[39]*.* Here, we used a mixed effects model to calculate intraclass correlation for single measurements per participant under different conditions; this is typically notated as ICC(3,1). This assesses test–retest reliability by isolating variance between participants and residual variance to quantify measurement consistency. Although there are no definitive guidelines for precise interpretation of ICCs, results have frequently been binned into four quality groups where 0.0–0.5 is “*poor*”, 0.50–0.75 is “*moderate*”, 0.75–0.9 is “*good*” and 0.9–1.0 is “*excellent*” [40].

#### Bland–altman metrics of reliability

In contrast to ICCs, Bland–Altman analyses investigate reliability by considering the differences between paired groups of measurements [41, 42]. We were interested in: (1) the mean difference between methods (chronological age—brain age), also known as “bias”; and (2) the bias ± 1.96 standard deviations of the differences, also known as “limits of agreement” (LoA). Narrower ranges of these limits indicate better agreement. Given that our data was non-normally distributed, we used nonparametric approaches that construct the limits using percentiles of the distribution rather than assuming normality. This was done via the R package *“SimplyAgree*” [43], separately for no versus low-motion and then no versus high-motion scans. For Bland–Altman metrics, there are no universal interpretations for bias and limits of agreement, instead being dependent on the context and desired accuracy [44]. While this research area is in a nascent state, we wanted to provide some broad guidelines related to these outcomes. We therefore reported bias, limits of agreement, and the percentage of predicted brain ages from each algorithm that were between − 3.55 and + 3.55 years (our chosen maximal allowable difference [MAD] for chronological—brain age). This threshold was derived from a recent mega-analysis that found this amount of brain age acceleration in patients with severe mental illness [45].

#### Effect size estimates of motion effects

We constructed linear mixed effect models that could accommodate repeated measures from the same individuals and examined the high-, low-, and no-motion conditions. To investigate the effect of movement condition on brain age, movement condition was input as a fixed effect and participant ID was included as a random effect to account for the repeated measures design. We then compared differences in brain age between the no movement and low movement conditions, and between the no movement and high movement conditions. This was completed with the *lme4* package in R. For these analyses, we calculated partial Eta^2^ as an effect size (ES) and binned these ES into four classes: ES < 0.02 as "very small”, 0.02 < ES < 0.13 as “small”, 0.13 < ES < 0.26 as “medium”, and ES > 0.26 as “large”.

#### Additional analyses

Complementing these quantitative metrics, we also wanted to depict the variability in predicted brain age and brain age delta for no-, low, and high-motion scans. To do this, we constructed repeated measure graphs (aka “spaghetti plots”) of differences in brain age (predicted age and brain age delta) across the different scan types. Additional analyses are detailed in our Supplemental Materials (Additional file 1) which include bivariate correlations between algorithms and comparison of image quality across moving and non-moving scans.

## Results

### Evaluation metrics for each algorithm

We calculated MAE and RMSE for each algorithm for each scan type (no-, low- and high-motion). As shown in Table 1, across algorithms, prediction errors increased with greater motion, with the largest errors observed for ENIGMA (MAE 9.967 to 11.549; RMSE 12.145 to 13.535) and the smallest errors for DeepBrainNet (MAE 3.497 to 4.019; RMSE 4.629 to 5.230) and pyment (MAE 3.139 to 3.326; RMSE 4.102 to 4.073). The brainageR, XGBoost, and ENIGMA algorithms showed substantial declines in performance with motion, while DeepBrainNet and pyment were more robust.

**Table 1:** Predictive performance metrics of five brain age algorithms (brainageR, DeepBrainNet, XGBoost, ENIGMA, pyment) across three motion conditions (no motion, low motion, high motion)

Predictive metrics (comparing chronological age and brain age)				
Algorithms		No motion	Low motion	High motion
brainageR	MAE	4.043	5.316	7.236
	RMSE	5.128	7.150	9.535
DeepBrainNet	MAE	3.497	3.937	4.019
	RMSE	4.629	5.121	5.230
XGBoost	MAE	6.927	7.642	9.021
	RMSE	9.025	9.647	10.757
ENIGMA	MAE	9.967	10.827	11.549
	RMSE	12.145	12.459	13.535
pyment	MAE	3.139	3.310	3.326
	RMSE	4.102	4.143	4.073

Performance was evaluated by mean absolute error (MAE) and root mean squared error (RMSE) between algorithm predicted brain age and chronological age

### Graphical depiction of variability in brain age delta

**Fig. 3:**
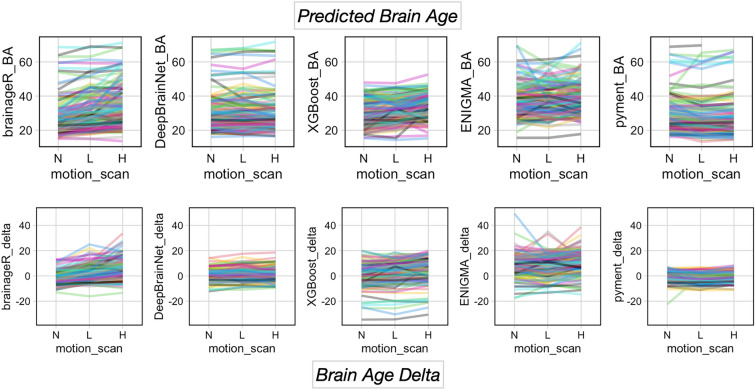
Repeated line graphs of predicted age (top) and brain age delta (bottom) for each algorithm. Motion level is shown on the horizontal axis with no motion on the left (N), low motion in the middle (L), and high motion on the right (H). The vertical axis is the predicted brain age or brain age delta (with equal axes for each graph). The order of the plots for each row from left to right is brainageR, DeepBrainNet, XGBoost; ENIGMA, and pyment

Complementing our calculation of evaluation metrics, we also wanted to depict the variability in predicted brain age and brain age delta for no-, low, and high-motion scans. To visualize differences in brain age across the different scan types, we have included repeated measure graphs (aka “spaghetti plots”) shown below in Fig. 3.

### Brain age reliability by algorithm, as assessed by intraclass correlations

We examined reliability using intraclass correlations (ICCs) for our five algorithms of interest, comparing no to low motion scans and no to high motion scans. For “raw” brain age, DeepBrainNet and pyment showed excellent reliability when comparing no motion to versus low- and also high- motion scans across all scan types (ICC 0.956–0.965 no vs low motion; 0.944–0.956 no vs high motion). These results are shown in Table 2. BrainageR XGBoost, and ENIGMA had lower ICCs indicating poorer reliability, particularly for high motion scans (brainageR ICC 0.712; ENIGMA ICC 0.709; XGBoost ICC 0.609). For brain age delta, XGBoost showed the highest reliability for no vs low motion scans (ICC 0.873), while DeepBrainNet demonstrated good reliability across both comparisons (ICC 0.825 and 0.788). BrainageR had the lowest ICCs, indicating less reliable brain age deltas, particularly for high motion (ICC 0.351). These results are shown in Table 3.

**Table 2:**
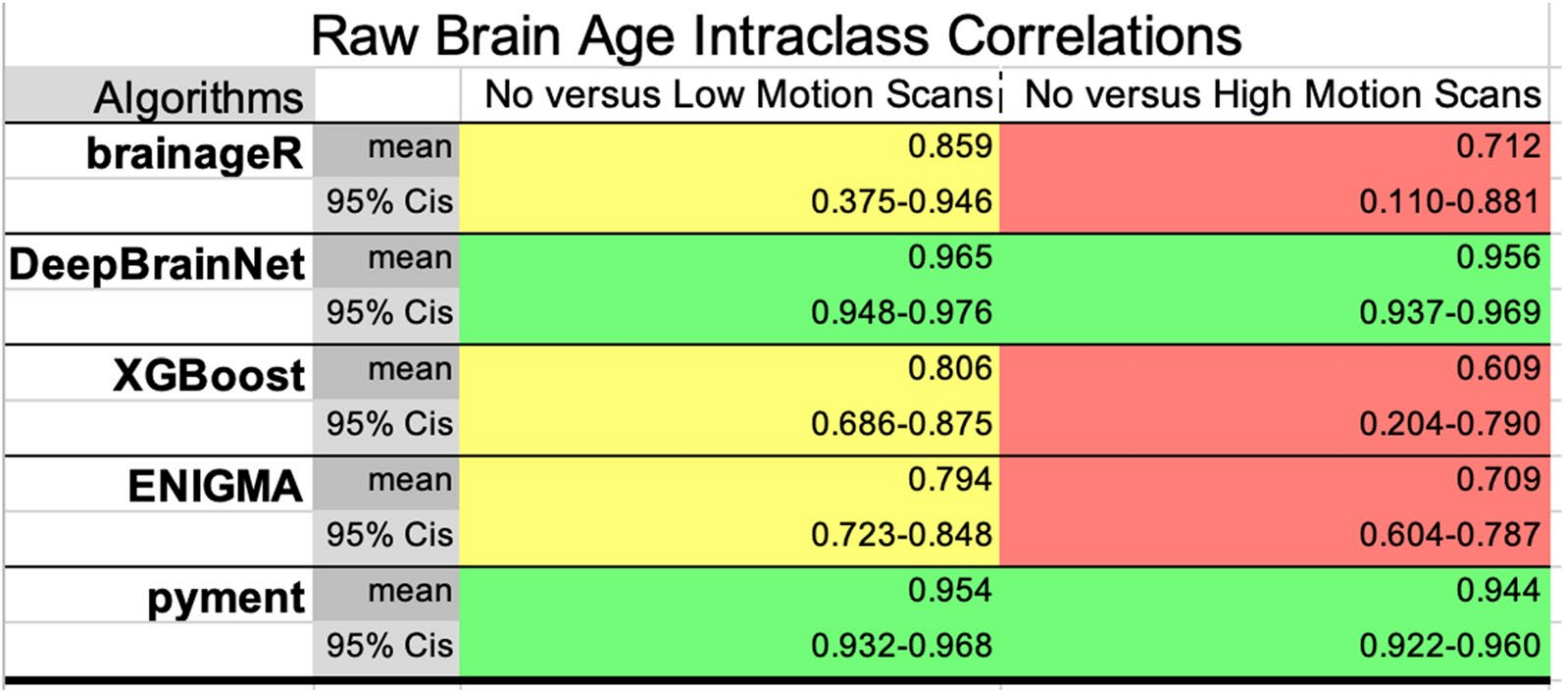
Intraclass correlation coefficients (ICCs) evaluating reliability of “raw” brain age from five algorithms (brainageR, DeepBrainNet, XGBoost, ENIGMA, pyment) between no motion and low motion scans, and between no motion and high motion scans ICC values quantify consistency of brain age outputs; higher values indicate greater reliability

Algorithms with ICCs in the “*poor*” (0.0–0.5) or “*moderate*” (0.50–0.75) ranges are colored in red, while those with “*good*” ICCs (0.75–0.9) are colored in yellow and “*excellent*” ICCs (0.9–1.0) are colored in green

**Table 3:**
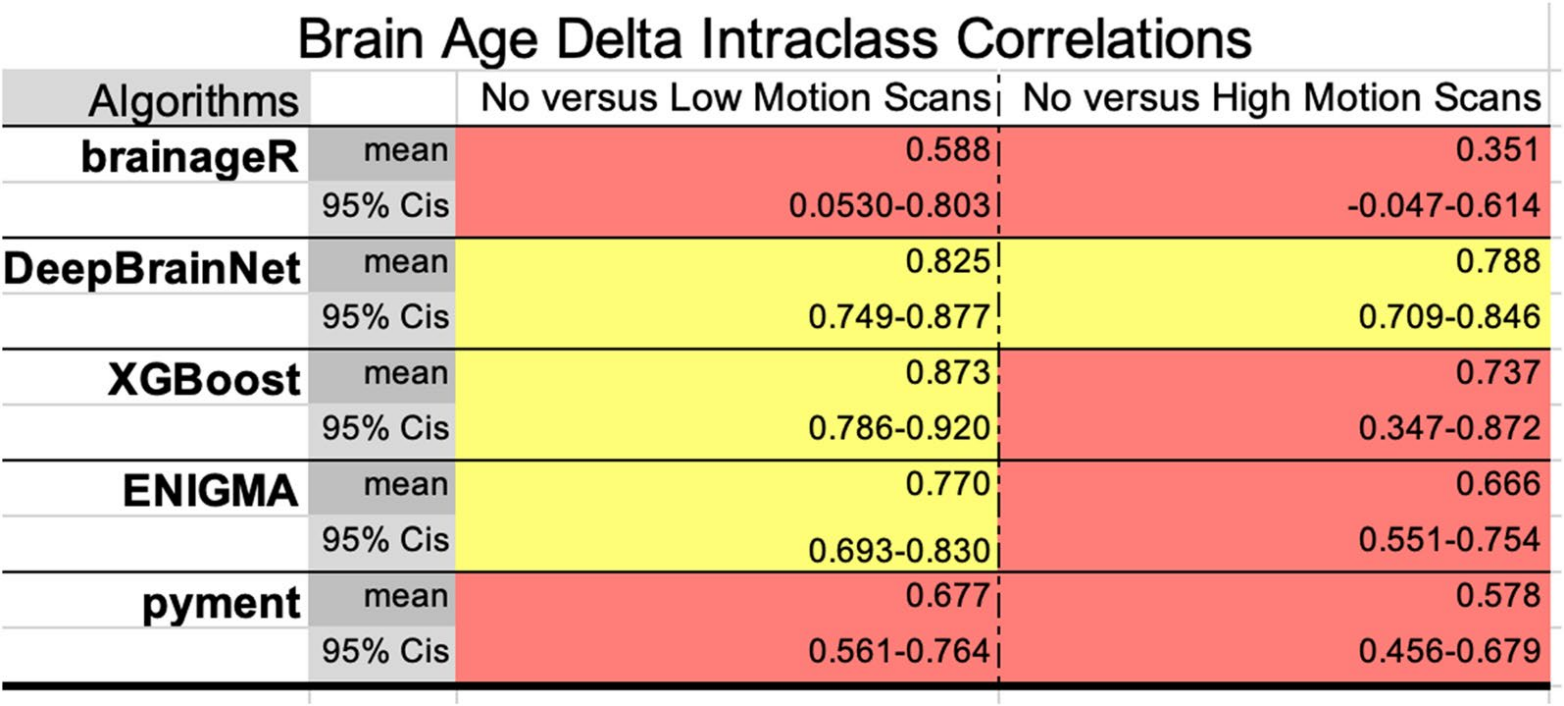
Intraclass correlation coefficients (ICCs) evaluating reliability of brain age delta values (differences between predicted and chronological age) from five algorithms (brainageR, DeepBrainNet, XGBoost, ENIGMA, pyment)

ICCs were calculated between no motion and low motion scans, and no motion and high motion scans. Higher ICC values indicate more reliable, consistent delta values across motion conditions. Algorithms with ICCs in the “*poor*” (0.0–0.5) or “*moderate*” (0.50–0.75) ranges are colored in red, while those with “*good*” ICCs (0.75–0.9) are colored in yellow

### Bland–altman metrics of reliability by algorithm

There was a high degree of variability when examining Bland–Altman metrics of reliability for each algorithm (as shown in Table 4). Of note, narrower LoAs and higher % within MAD indicates better agreement. DeepBrainNet and pyment showed the best agreement, with tight LoAs and high % within MAD across motion conditions. XGBoost and ENIGMA had wider LoAs and lower % within MAD, reflecting poorer agreement. BrainageR demonstrated the lowest agreement for high motion scans (LoA − 24.254 to − 1.418; % within MAD 34.06%). While there are no defined cutoffs for MAD, it is notable that for multiple algorithms, a bare majority, or less, of the predicted brain ages are within this span (− 3.55 and + 3.55 years for predicted brain age—chronological age). This was seen for brainageR (for no versus low; and no versus high motion scans), ENIGMA (no versus high motion scans), and XGBoost (no versus high motion scans).

**Table 4:** Bland–Altman analysis evaluating agreement between brain age predictions from five algorithms (brainageR, DeepBrainNet, XGBoost, ENIGMA, pyment) for no motion versus low motion scans, and no motion versus high motion scans

Raw brain age bland altman analyses			
Algorithms		No versus low motion scans	No versus high motion scans
brainageR	Bias	− 3.682	− 5.410
	LoA	− 14.182–1.142	− 24.254–1.418
	% within MAD	48.55%	34.06%
DeepBrainNet	Bias	− 0.721	− 0.816
	LoA	− 6.9002–4.7241	− 6.4133–7.7052
	% within MAD	84.780%	77.540%
XGBoost	Bias	− 1.370	− 3.577
	LoA	− 12.389–5.976	− 17.542–7.398
	% within MAD	65.220%	44.930%
ENIGMA	Bias	− 0.689	− 1.553
	LoA	− 10.453–10.932	− 17.317–12.256
	% within MAD	64.490%	52.170%
pyment	Bias	0.805	0.631
	LoA	− 2.3343–6.9135	− 5.8886–7.7195
	% within MAD	87.680%	85.510%

Results include the mean bias, 95% limits of agreement (LoA), and percentage of points within the margins of agreement (% within MAD)

### Statistical quantification of participant motion, as assessed by linear mixed effects models

Using linear mixed effects models with repeated measure, we calculated effect sizes, compared differences in brain age between the no movement and low movement conditions, and between the no movement and high movement conditions. This was completed with the *lme4* package in R. Overall, participant head motion had significant, though mostly modest impacts on raw brain age calculated by three of our algorithms, specifically DeepBrainNet, pyment, and ENIGMA. For DeepBrainNet, the overall effect was (*F(2,*283.73) = 4.445, p = 0.01, partial Eta^2^ = 0.03). For this algorithm, both low and high levels of motion scans had small effects on raw brain age calculation (low motion β = 0.06, high motion β = 0.06). For pyment, the overall effect was (*F(2,*283.81) = 7.637, p < 0.005, partial Eta^2^ = 0.05). For this algorithm, both low and high levels of motion scans had small negative effects on raw brain age calculation (low motion β = − 0.08, high motion β = − 0.03). With ENIGMA, there was an increasing but still modest effect of motion (*F(2,*283.37) = 8.526, p < 0.005, partial Eta^2^ = 0.06). For this algorithm, low motion had a small effect on raw brain age calculations, while high motion had a significantly larger, albeit modest, effect (low motion β = 0.09, high motion β = 0.22). Effects of motion were more pronounced for XGBoost and brainageR. ANOVAs indicated large effects for motion for both of these algorithms (XGBoost *F(2,*283.99) = 58.008, p < 0.005, partial Eta^2^ = 0.29; brainageR *F(2,*283.35) = 111.65, p < 0.005, partial Eta^2^ = 0.44). For these algorithms, both low and high levels of motion scans had moderate effects on raw brain age calculation (XGBoost low motion β = 0.25, high motion β = 0.57; brainageR low motion β = 0.35, high motion β = 0.55).

For brain age delta, effects were larger in magnitude, but algorithmic performance mostly mirrored results for raw brain age. Omnibus tests were nearly identical but probing of low and high levels of motion scans (compared to no motion scans) revealed some subtle differences in effects. For DeepBrainNet, both low and high levels of motion scans had small effects on brain age delta (low motion β = 0.14, high motion β = 0.14). Effects were very similar for ENIGMA brain age delta (low motion β = 0.11, high motion β = 0.24). For pyment, different levels of motion had negative effects, though with similar absolute magnitudes (low motion β = − 0.23, high motion β = − 0.09). Similar to raw brain age, effects were largest for low and high levels of motion for XGBoost and brainageR brain age delta (XGBoost low motion β = 0.20, high motion β = 0.43; brainageR low motion β = 0.61, high motion β = 0.94).

## Discussion

In this study, we examined the effect of movement during MRI scans on calculations of brain age using multiple, well-validated, and commonly deployed calculation algorithms. Given that motion artifacts can skew volumetric and morphological measures used to determine brain age, it is critical to track variations in the sensitivity of different brain age algorithms to these issues. To these ends, we used two measures of prediction (MAE; RMSE) and two metrics of reliability (ICCs; Bland Altman metrics) to compare the different algorithms, leading to multiple important findings. First, predictive errors increased with greater motion for all algorithms, but DeepBrainNet and pyment were most robust, maintaining low errors even for high motion scans (MAE 3–4 years). In contrast, ENIGMA showed substantially worse performance for motion scans (MAE up to 11.5 years). These are similar to past estimates from other groups focused on evaluation of brain age prediction [46]. Using ICCs for brain age, DeepBrainNet and pyment demonstrated excellent reliability for brain age predictions, with high ICCs of 0.956–0.965 for no vs low motion and 0.944–0.956 for no vs high motion scans. The other algorithms showed poorer reliability, particularly XGBoost and brainageR where ICCs dropped as low as 0.609 for high motion scans. Turning to Bland Altman metrics, two algorithms, DeepBrainNet and pyment, had a small degree of bias for raw brain age, tighter LoA, and higher % of predicted points within a predefined maximum allowable difference. Performance was a bit poorer for the ENIGMA and XGBoost algorithms, and the brainageR algorithm showed the largest amount of bias when examining motion and non-motion scans. Critically, all the algorithms had sizable ranges of differences, indicating that participant motion could significantly influence brain age calculation for any given participant.

In addition to reliability analyses, we also constructed linear-mixed effect models to get specific statistical assessments of how much participant motion could influence brain age calculation. In these analyses, participant head motion had significant, though mostly modest impacts, on brain age calculations for three of our algorithms (specifically DeepBrainNet, pyments, and ENIGMA). Effects of motion were more pronounced for XGBoost and brainageR, with these statistical models suggesting larger effects (XGBoost partial Eta^2^ = 0.29; brainageR partial Eta^2^ = 0.44). Overall, our findings demonstrate that motion during MRI scanning can significantly influence brain age predictions depending on the algorithm used. However, this impact is not consistent across all methods. The brainageR algorithm may be less desirable for expanded deployment, while DeepBrainNet and pyment may have greater noise tolerance. ENIGMA and XGBoost performances were more average and should be explored in greater depth.

Digesting our results, there is some divergence in ICCs for raw brain age and brain age delta and this is likely due to differences in interindividual variance across the measures. Specifically, ICCs assess reliability by comparing interindividual variance to total variance. For raw brain age, this interindividual variance reflects differences in predicted biological/brain ages between participants; however, and in contrast to brain age delta, the interindividual variance is slightly reduced as actual age is subtracted from raw brain age. This leaves variance primarily due to prediction error. As such, lower, interindividual variance may be expected for brain age delta, potentially driving down ICC values. However, by accounting for actual age, brain age delta may isolate prediction error and provide a more conservative estimate of brain-based age prediction reliability. This is something to consider as more research groups consider brain age algorithm development and benchmarking [47, 48].

### Relating our findings to past brain age publications

Thinking about our findings in relation to past reports, similar patterns have been noted individually for each algorithm regarding reliability and relations with other critical variables (i.e., age; image quality). When examining raw brain age, there were reasonably high correlations between the 5 different algorithms we investigated with r’s ranging from 0.67–0.93 (as noted in Additional file 1). Of note, we deployed brain age algorithms that used NIfTI files processed by the algorithm’s code (i.e., brainageR; DeepBrainNet), as well as outputs from Freesurfer. Our results also clearly connect to past work finding variations in morphometric values derived from high motion scans. Such effects remain after different forms of manual and automatic correction, suggesting that in-scanner motion induces spurious effects that do not reflect a processing failure in software; rather, they reflect systematic bias (e.g., motion-induced blurring) and this may appear similar to gray matter atrophy. Particularly concerning, many neuroimaging groups will visually inspect scans and include scans of “fair” or “marginal” quality. As researchers focus on different populations (e.g., children versus adolescents; clinical groups versus non-clinical groups), this potentially creates an “apples versus oranges” comparison; all scans may “pass” visual inspection, but one group has excellent image quality and clarity, while another has visible motion and is only above these passing thresholds.

Regarding the impact of participant motion, past work may underestimate the true impact of motion and noise in brain age calculation. Work by past investigators has found between-person relations between image quality and brain age calculation. Our project, however, is the first to examine intra-individual (within-subject) differences. The use of repeated MRI scans from the same participants allows us to control for confounding variables due to individual differences and understand relationships between motion artifacts and brain age calculation. By utilizing a within-person design with multiple scans per person over time, we isolated the effects of scan quality while holding constant time-invariant factors. This improves our understanding about these effects, as we are separating between- and within-person sources of variation.

Connected to this, our team is particularly interested in the effects of image quality and motion on brain age calculation. To our knowledge, no brain age algorithms have integrated measures of image quality in their model training and testing. In future development of brain age algorithms, it would be interesting to examine whether measures of image quality and successful preprocessing (i.e., CAT12 grades, or Freesurfer’s Euler Number) could be used to further optimize models. Certain brain age models (i.e., pyment) have used large numbers of participants (N = 53,542) in their algorithmic development. This has meant that a large number of high motion participants have been included in training and test datasets. While this may mean less error when dealing with high-motion scans, image quality was not explicitly modeled. Given that commonly used structural MRI measures derived from T1-weighted images are strongly related to image quality, this could be a fruitful future direction. Tackling these and other open questions related to brain age could significantly advance our understanding of healthy, as well as accelerated, aging processes.

### Limitations of the current project

While we believe we advanced applied understanding of brain age calculation, our work is not without limitations. First, our data is cross-sectional in nature, and it will be important to think about estimation and validation of different performance metrics in participants with repeated MRI scans separated by long periods of time. By looking longitudinally at within- and between-person change in relation to different algorithms, we may be able to derive a particularly powerful window into age-associated functional declines and disease, and different clinically relevant issues. It would be particularly powerful if there were high, low, and no motion MRI acquisitions acquired longitudinally to richly probe these questions. Second, we tested five commonly used algorithms where code was publicly shared for mass implementation of brain age calculation. There are many in-press and preprinted manuscripts engineering new calculations of brain age. Such novel algorithms may exhibit superior performance and fewer limitations than the approaches we examined here. It would be useful for novel algorithms to reuse this dataset to compare performance to what is reported here and demonstrate relative superiority. Third, we did not connect variations in brain age at different levels of movement with behavioral phenotypes of interest. In past work, we found that XGBoost brain age calculations, compared to those derived from brainageR and DeepBrainNet, were more sensitive to the detection of clinical diagnoses of cognitive impairment [19]. We, however, did not calculate pyment or ENIGMA brain ages in that past project. It would be clinically useful to examine if brain age calculated from scans at various motion levels were sensitive to clinical characteristics commonly investigated in brain age research studies (e.g., Alzheimer’s Disease; schizophrenia). The sample here was healthy, sampled primarily in early adulthood (Mean Age = 30.01), but without dense sampling of relevant psychological or neurological variables. Examined collectively, it will be important for this subfield of neuroimaging to show that brain age algorithms are reliable, even with variable levels of motion, and that algorithms identify unique and additive variance in brain age.

A connected, potential limitation is how motion was induced in participants (i.e., directing participants to nod in the scanner). While nodding has commonly been reported as the most common form of motion in past MRI projects, explicitly directing participants may not fully reflect motion in the “real world”. This project did not have direct measurements of movement. Past research groups have noted that the primary drivers of participant motion may be a combination of nodding, relaxation of the participant’s neck muscles, and compression of the foam padding which the participant’s head lies on. However, multiple groups have noted that problematic head motion is “*composed of a single type of biomechanical motion, which we infer to be a nodding movement*” [21] and “typically produced by z-axis displacement (e.g., nodding)” introducing artifacts in f/MRI data [26]. The high motion scans had more occurrence of movement (related to the instructions given to participants), but (low or high) motion scans could have variability on the range, frequency, and type of motion that participants engaged in. Deeper investigations on this topic are warranted, especially in clinical settings, as differences in participant instructions, compliance, and the ability to practice or complete mock MRI scans may help us generalize our findings out to high-motion, pediatric and clinical populations.

Limitations notwithstanding, additional research on “brain age” is imperative. Richer information about the brain and brain aging could be important for those focused on age-related mortality and morbidity. Of note, given that these algorithms are often easy to implement, it could be advantageous for the field to report group comparisons or behavioral correlations with multiple algorithms. Typically, a research group will simply deploy a single algorithm and report results; it is often unclear if results would generalize with different algorithmic derivations of brain age and brain age delta. However, thoughtful consideration about reliability and noise tolerance will be critical when making decisions about different brain age algorithms, especially with an ever-growing landscape of potential ways to calculate this variable. Evaluating model performance on datasets with controlled motion artifacts can better establish the validity of brain age as a predictive measure in aging research. Moving forward, further optimization and validation of brain age algorithms is needed to ensure clinical utility and reliability of this biomarker.

## Conclusions

This study systematically examined the effects of participant motion on brain age predictions from five popular algorithms using a dataset with repeated scans of varying motion levels. Results demonstrated that motion significantly impacted brain age estimates for some algorithms, with intraclass correlations and errors increasing with greater motion. DeepBrainNet and pyment showed the greatest robustness to motion effects, maintaining high reliabilities and smaller errors across conditions. In contrast, XGBoost and brainageR exhibited larger errors and lower reliabilities with motion. Objective image quality assessments confirmed differences in motion levels between scans. Overall, this study provides empirical evidence that motion artifacts can influence brain age calculations, with implications for algorithm selection and reliability when head motion may be present.

### Supplementary Information

**Additional file 1.** Supplemental Materials with Additional Analyses.

## Data Availability

This dataset is publicly available at OpenNeuro at: https://openneuro.org/datasets/ds004173/
