# Supplementary material for: Examining the reliability of brain age algorithms under varying degrees of participant motion
Source: Brain Inform. 2024 Apr 4;11(1):9. doi: 10.1186/s40708-024-00223-0 (PMC10994881; doi:10.1186/s40708-024-00223-0)
Supplement: Supplementary file 1 — Additional file 1. Supplemental Materials with Additional Analyses. [file 40708_2024_223_MOESM1_ESM.docx]

**SUPPLEMENTAL MATERIALS**

**Bivariate Correlations Between Algorithms**

Paralleling past reports [1, 2] and to understand relations between different brain age algorithms, we first computed bivariate correlations between each of the algorithms for raw brain age, as well as the brain age delta (i.e., raw brain age - a participant’s chronological age). When examining raw brain age, there were reasonably high correlations between the 5 different algorithms we investigated with r’s ranging from 0.67-0.93 (as shown in Figure S1). For the brain age gap, these correlations were lower (range of r’s = 0.37-0.78), but still statistically significant (all p’s <.005). Interestingly, correlations were low between each algorithm’s raw brain age and brain age delta (max r, within algorithm=0.11, as shown in Figure S2).

**Figure S1.**


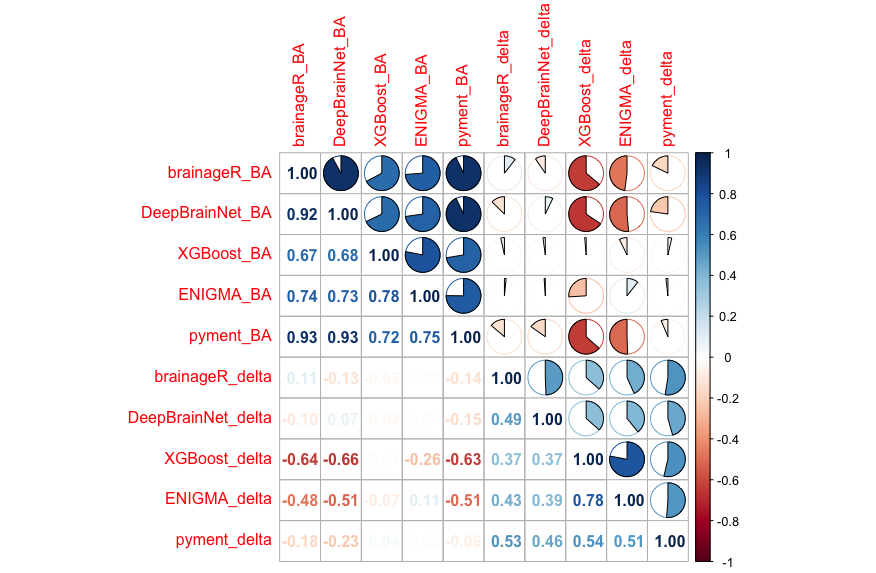


**Figure S1**. Correlation plot for algorithms of interest, for raw brain age and brain delta. The bottom left triangle shows the numeric values for the correlations, while the top right corner depicts the same relations using pie charts.

**Figure S2.**


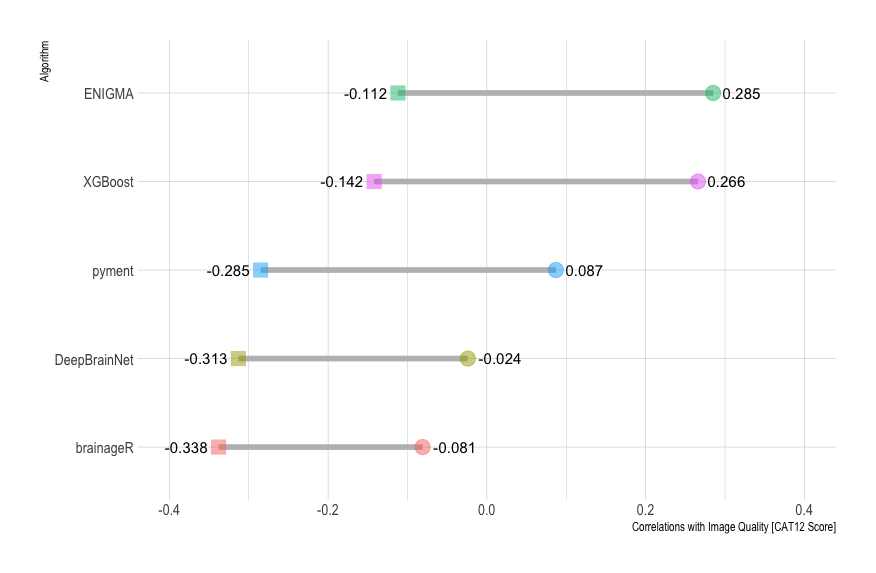


**Figure S2**. Correlation between image quality and brain age variables. Raw brain age is shown on the right side (depicted with circles) and brain age delta is shown on the left side (depicted with squares). The vertical axis shows each algorithm, while the horizontal axis depicts the magnitude of the correlation. Each algorithm is depicted with different colors (ENIGMA = light green; XGBoost = light purple; pyment = light blue; DeepBrainNet = light yellow; brainageR = light red).

**Comparison of Image Quality Across Moving and Non-Moving Scans.**

As noted in the main manuscript, participants were instructed to lay still, move their heads slightly, or move their heads in large amounts. When comparing image quality across different motion levels, as derived from the CAT12 toolbox, we found that moving scans had lower image quality than still scans (*F(2,285.6)*=253.82, p<.005, partial Eta^2^=0.64; within group differences are shown in Figure 3). This was a simple confirmation that motion during scans led to lower image quality. We also examined relations between image quality and each of the algorithms (for raw brain age and brain age delta). There were modest negative correlations, ranging from r=-0.112 to r=-0.338 between raw brain age and image quality. As such, lower quality scans were related to higher raw brain age values. Image quality was positively and negatively correlated with brain age delta, depending on the algorithm of interest (r range = -0.081 to 0.285 shown in Figure S3).

**Figure S3.**


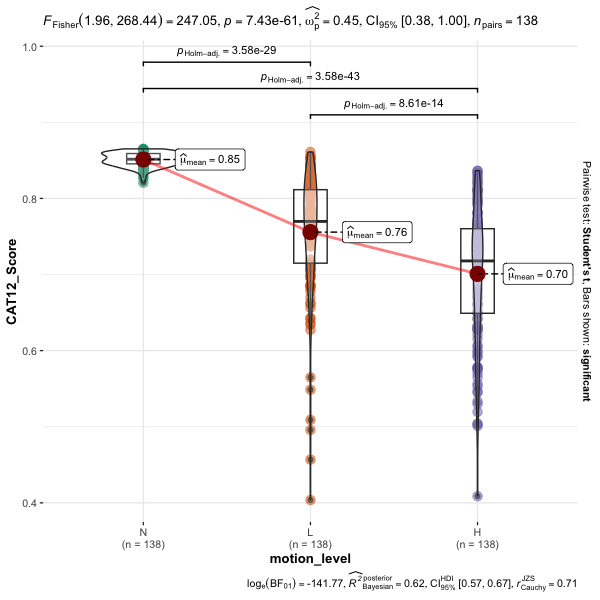


**Figure S3.** Comparison of image quality (CAT12_Scores) across movement conditions. Boxplots showing distributions of CAT12 image quality scores for no motion, low motion, and high motion scans. Higher scores indicate better image quality. There were significant differences between no and low motion scans, low and high motion scans, and no and high motion scans.

**SUPPLEMENTAL REFERENCES**

1. Bacas E, Kahhalé I, Raamana PR, et al (2023) Probing multiple algorithms to calculate brain age: Examining reliability, relations with demographics, and predictive power. Hum Brain Mapp

2. Dörfel RP, Arenas-Gomez JM, Fisher PM, et al (2023) Prediction of brain age using structural magnetic resonance imaging: A comparison of accuracy and test-retest reliability of publicly available software packages. BioRxiv 2023–01
